# A Contribution to the Physiology of the Ureter and Vas Deferens

**Published:** 1917-04

**Authors:** D. I. Macht


### A Contribution to the Physiology of the Ureter and Vas Deferens.

BY D. I. MACHT.

Studies were made on the siolated ureter and vas deferens of animals and from the surgical operating room, and were confirmed by observations of those organs in situ in various animals. The isolated ureter is best studied by means of ureteral rings. These contract rhythmically so that the rate and force of peristaltic movements and the tonus of the ureter can be studied. The optimum medium is a Locke solution plus a small quantity of fresh urine. Urea stimulates the contractions of the ureter; a slightly acid medium is also necessary for the furtherance of the contractions. The vas deferens, on the contrary, survives best in a slightly alkaline medium. These conditions of acidity and alkalinity correspond to those in nature. Oxygen is necessary for the proper maintenance of the contractions of both ureter and vas deferens. Heat first stimulates and subsequently paralyzes the contractions. Cold slowly inhibits them.

Both ureter and vas react to epinephrin, which fact proves that they are inervated by the true sympathetic. The response to ergotoxin still further corroborates this fact.

Both ureter and vas react to the socalled parasympathetic drugs: pilocarpin, physostigmin, cholin, muscarin and atropin, which fact proves that they are also inervated by the parasympathetic fibers.

Both ureter and vas react to nicotin, which fact points to the presence of ganglion cells in their walls.

---

Pellagra may be prevented or cured by proper diet.

---

The United States Public Health Service believes that the common towel spreads trachoma, a disease of the eyes.

